# Interaction between neuronal calcium sensor protein 1 and lithium in pedunculopontine neurons

**DOI:** 10.14814/phy2.13246

**Published:** 2017-04-13

**Authors:** Stasia D'Onofrio, James Hyde, Edgar Garcia‐Rill

**Affiliations:** ^1^ Center for Translational NeuroscienceDepartment of Neurobiology and Developmental SciencesUniversity of Arkansas for Medical SciencesLittle RockArkansas; ^2^ Department of Psychiatry and Center for Neural Basis of CognitionUniversity of PittsburghPittsburghPennsylvania

**Keywords:** Arousal, bipolar disorder, gamma oscillations, sleep‐wake

## Abstract

Bipolar disorder is characterized by sleep dysregulation, suggesting a role for the reticular activating system (RAS). Postmortem studies showed increased expression of neuronal calcium sensor protein 1 (NCS‐1) in the brains of some bipolar disorder patients, and reduced or aberrant gamma band activity is present in the same disorder. Lithium (Li^+^) has been shown to effectively treat the mood disturbances in bipolar disorder patients. We previously showed that NCS‐1 at low levels increased, and at high levels decreased, gamma oscillations in RAS pedunculopontine neurons (PPN), and that Li^+^ decreased these oscillations. We previously described the effects of each agent on oscillations, G‐protein mechanisms, and Ca^2+^ currents. However, we designed the present experiments to determine the nature of the interaction of NCS‐1 and Li^+^ at physiological concentrations that would have an effect within minutes of application. As expected, Li^+^ decreased gamma oscillation amplitude, while NCS‐1 increased the amplitude of gamma oscillations. We identified NCS‐1 at 2 μmol/L as a concentration that increased gamma oscillations within 5–10 min, and Li^+^ at 10 μmol/L as a concentration that decreased gamma oscillations within 5 min. The combined application of NCS‐1 and Li^+^ at these concentrations showed that Li^+^ reduced the effects of NCS‐1 on oscillation amplitude within 5–10 min. These results demonstrate that at physiological levels, Li^+^ acts to reduce the effects of NCS‐1 so that, given over expression of NCS‐1, Li^+^ would have salutary effects.

## Introduction

Human postmortem studies reported increased expression of high affinity, low capacity neuronal calcium sensor protein 1 (NCS‐1) in the brains of some bipolar disorder and schizophrenic patients (Koh et al. 2003). Reduced or aberrant gamma band activity has been reported in the same disorders (Ozerdem et al. 2011; Uhlhaas and Singer 2010). That is, gamma band activity is disrupted in the same disorders that show over expression of NCS‐1 (Senkowski and Gallinat 2015; Wynn et al. 2015). Cognitive and executive functions are associated with gamma‐band activity (Eckhorn et al. 1988; Gray and Singer 1989; Singer 1993; Philips and Takeda 2009). Additionally, gamma oscillations are essential to information processing during sensory perception, motor behavior, and memory formation (Kann et al. 2014), and are critical for communication among brain areas, thus allowing large‐scale integration of distributed sets of neurons (Rodriguez et al. 1999; Whittington et al. 2000; Nikolic et al. 2013). Large disturbances in neurocognition can be seen throughout the different stages of bipolar disorder. Symptoms of both manic and depressive episodes in bipolar disorder include sleep and circadian rhythm disturbances, emotional dysregulation, and cognitive impairment (Leboyer and Kupfer 2010). Specifically, attention and memory deficits, impairment in verbal recall and fine motor skills, and disturbance of sustained attention are present during depressive episodes, whereas during mania episodes dysfunctions are seen in attention, complex processing, memory, and emotional processing (Goldberg and Chengappa 2009). Cognitive deficits are present even during euthymia, where executive function, verbal memory, sustained attention, visual memory, and verbal fluency are disturbed (Bora et al. 2009). Considering that bipolar disorder is characterized by prominent sleep dysregulation, this indicates a role for the reticular activating system (RAS).

In a previous study examining the pedunculopontine nucleus (PPN), part of the RAS controlling waking and REM sleep, we found that NCS‐1 modulated Ca^2+^ channels in PPN neurons that generate gamma band oscillations, and that excessive levels of NCS‐1, as expected with over expression, reduced gamma band oscillations in these cells (D'Onofrio et al. 2015). We found 1 *μ*mol/L NCS‐1 to be the lowest concentration for gamma oscillation modulation, but its effects showed a long latency ~20 min. Li^+^ at high concentrations (1–10 mmol/L) had a short latency effect in inhibiting oscillations. These results suggested that NCS‐1 over expression may be responsible for the decrease in gamma band activity present in at least some bipolar disorder patients, and that Li^+^ may counteract its effects. Li^+^ has been shown to act by inhibiting the interaction between NCS‐1 and inositol 1,4,5‐triphosphate receptor protein (InsP_3_R) (Schlecker et al. 2006). NCS‐1 is known to enhance the activity of InsP_3_Rs (Kasri et al. 2004), thus amplifying the Ca^2+^ signal through these receptors. Importantly, InsP_3_Rs are present in the PPN (Rodrigo et al. 1993).

The goal of this study was to determine the nature of low concentration, short latency (within 5 min) effects of NCS‐1 and Li^+^ in the PPN, and to identify the short latency interaction between Li^+^ and NCS‐1 at physiological concentrations in the micromolar range. Since Li^+^ has been shown to act by inhibiting NCS‐1/InsP_3_R interaction, we hypothesize that Li^+^ will reduce the effects of over expression of NCS‐1, therefore preventing the down regulation of gamma band activity and restoring normal levels of gamma oscillations. Our findings provide a novel area of future research to determine if this intracellular mechanism is involved in the treatment of mood disturbances seen in bipolar disorder patients, and point to new therapeutic targets for alleviating some of the arousal and sleep/wake disturbances in this devastating disease.

## Methods and Materials

### Slice preparation

Pups aged 9–13 days of either sex from adult timed‐pregnant Sprague‐Dawley rats (280–350 g) were anesthetized with ketamine (70 mg/kg, I.M.) until tail pinch reflex was absent. This age range was selected due to the developmental decrease in REM sleep of the rat that occurs between 10 and 30 days (Jouvet‐Mounier et al. 1970). This period of investigation enabled sampling from a baseline period (9–13 days), before the epoch of the greatest transitions that peak at 14–16 days and continue until >20 days, as determined by our previous body of work on the PPN (Garcia‐Rill et al. 2008). Pups were decapitated and the brain was rapidly removed and cooled in oxygenated sucrose‐artificial cerebrospinal fluid (sucrose‐aCSF). The sucrose‐aCSF consisted of (in mmol/L): 233.7 sucrose, 26 NaHCO_3_, 3 KCl, 8 MgCl_2_, 0.5 CaCl_2_, 20 glucose, 0.4 ascorbic acid, and 2 sodium pyruvate. Sagittal sections (400 *μ*m) containing the PPN were cut and slices were allowed to equilibrate in normal aCSF at room temperature for 1 h. The aCSF was composed of (in mmol/L): 117 NaCl, 4.7 KCl, 1.2 MgCl_2_, 2.5 CaCl_2_, 1.2 NaH_2_PO_4_, 24.9 NaHCO_3_, and 11.5 glucose. Slices were recorded at 37°C while perfused (1.5 mL/min) with oxygenated (95% O_2_–5% CO_2_) aCSF in an immersion chamber for patch clamp studies as previously described (Kezunovic et al. 2011, 2012, 2013). The superfusate contained the following synaptic receptor antagonists: the selective NMDA receptor antagonist 2‐amino‐5‐phosphonovaleric acid (APV, 40 μmol/L), the competitive AMPA/kainate glutamate receptor antagonist 6‐cyano‐7‐nitroquinoxaline‐2,3‐dione (CNQX, 10 μmol/L), the glycine receptor antagonist strychnine (STR, 10 μmol/L), the specific GABA_A_ receptor antagonist gabazine (GBZ, 10 μmol/L), and the nicotinic receptor antagonist mecamylamine (MEC, 10 μmol/L), collectively referred to here as synaptic blockers (SBs). All experimental protocols were approved by the Institutional Animal Care and Use Committee of the University of Arkansas for Medical Sciences and were in agreement with the National Institutes of Health guidelines for the care and use of laboratory animals.

### Whole‐cell patch‐clamp recordings

Differential interference contrast optics was used to visualize neurons using an upright microscope (Nikon FN‐1, Nikon, USA). Whole‐cell recordings were performed using borosilicate glass capillaries pulled on a P‐97 puller (Sutter Instrument Company, Novato, CA) and filled with a high‐K^+^ intracellular solution, designed to mimic the intracellular electrolyte concentration, of (in mmol/L): 124 K‐gluconate, 10 HEPES, 10 phosphocreatine di tris, 0.2 EGTA, 4 Mg_2_ATP, 0.3 Na_2_GTP; or a high‐Cs^+^/QX‐314 intracellular solution (in mmol/L): 120 CsMeSO_3_, 40 HEPES, 1 EGTA, 10 TEA‐Cl, 4 Mg‐ATP, 0.4 mmol/L GTP, 10 Phosphocreatine, 2 MgCl_2_. NCS‐1 (Prospec, Ness‐Ziona, Israel) was dissolved in the intracellular solution needed to perform each set of experiments at the concentrations described in the Results, as previously described (D'Onofrio et al. 2015). All recording electrodes had 1.2 *μ*L of standard intracellular solution injected at the tip, and the remainder of the pipette was filled with the solution (18–20 *μ*L) of the concentration of NCS‐1 to be tested. This allowed control recordings to be obtained soon after patching, while also allowing NCS‐1 to diffuse into the cell. The large volume in the pipette ensured that the concentration to which the cell was exposed was stable throughout the recording period, attaining steady‐state levels of NCS‐1 for >30 min (D'Onofrio et al. 2015). Osmolarity was adjusted to ~270–290 mOsm and pH to 7.3. The pipette resistance was 2‐5 MΩ. All recordings were made using a Multiclamp 700B amplifier (Molecular Devices, Sunnyvale, CA) in both current and voltage clamp mode. Digital signals were low‐pass filtered at 2 kHz, and digitized at 5 kHz using a Digidata‐1440A interface and pClamp10 software (Molecular Devices).

The recording region was located mainly in the *pars compacta* in the posterior PPN, immediately dorsal to the superior cerebellar peduncle. This area of PPN has been shown to have the highest density of cells (Wang and Morales 2009; Ye et al. 2010). Gigaseal formation and further access to the intracellular neuronal compartment was achieved in a voltage‐clamp configuration mode, setting the holding potential at −50 mV (i.e., near the average resting membrane potential of PPN neurons (D'Onofrio et al. 2015; Kezunovic et al. 2011, 2013). Within a short time after rupturing the membrane, the intracellular solution reached equilibrium with the pipette solution without significant changes in either series resistance (ranging 4–13 MΩ) or membrane capacitance values.

To study subthreshold oscillations of PPN neurons, whole‐cell patch‐clamp configuration was switched to current‐clamp mode. Average resting membrane potentials and bridge values in current clamp were 55 ± 2 mV and 11 ± 2 MΩ, respectively (*n* = 27). PPN cell type I PPN neurons (LTS current), type II PPN cells (Ia current), and type III PPN neurons (LTS + Ia currents) were identified as previously described (Kezunovic et al. 2011, 2012, 2013). All these cell types manifested gamma band oscillations when membrane potential was depolarized using a 1 sec duration ramp current clamp protocol (Kezunovic et al. 2011, 2012, 2013).

### Drug application

Bath‐applied drugs such as SBs were administered to the slice via a peristaltic pump (Cole‐Parmer, Vernon Hills, IL), and a three‐way valve system such that solutions reached the slice 1.5 min after the start of application. The Na^+^ channel blocker TTX, and the SBs were purchased from Sigma Aldrich (St. Louis, MO). Cholinergic antagonists were purchased from Sigma Aldrich, mecamylamine (MEC, a nicotinic receptor antagonist), as well as tetraethylammonium (TEA, a wide‐range K^+^ channel blocker). NCS‐1 (human recombinant) was purchased from Prospec Protein Specialist (Ness‐Ziona, Israel). The effects of NCS‐1 on single cell oscillatory activity were studied by allowing passive diffusion of NCS‐1 (with 1.2 μL of standard intracellular solution first loaded into the pipette tip, followed by 18–20 μL of the concentration of NCS‐1 to be tested) intracellularly through the recording micropipette, during extracellular superfusion of synaptic blockers, channel blockers, and TTX in aCSF extracellular solution.

### Data analysis

Off‐line analyses were performed using Clampfit software (Molecular Devices, Sunnyvale, CA). Comparisons between groups were carried out using a one‐way ANOVA, with Bonferroni post hoc testing for multiple comparisons. As stated above, we used 1 sec duration ramps applied every 5 min in current clamp in the presence of SBs and TTX to record membrane oscillations in all three PPN cell types. Peak oscillatory amplitude was analyzed by first filtering each ramp recording and measuring the three highest amplitude oscillations to derive a mean amplitude induced during each ramp. F values and degrees of freedom are reported for all linear regression ANOVAs. Differences were considered significant at values of *P* ≤ 0.05. All results are presented as mean ± SE.

## Results

Whole‐cell patch clamp recordings were performed in a total of 27 single PPN neurons to assess the effect of NCS‐1 and Li^+^ on intrinsic membrane properties, as well as to determine whether Li^+^ would affect the action of NCS‐1 at short latency. The neurons were localized in the *pars compacta* in the posterior PPN, which is easily identified in sagittal sections of the brainstem (Simon et al. 2010; Kezunovic et al. 2011). We first identified PPN neurons by cell type as previously described (Garcia‐Rill et al. 2007, 2008; Simon et al. 2010). No difference in average resting membrane potential was observed among PPN neuronal types. We previously showed that, regardless of cell type, voltage‐dependent, high threshold N‐ and P/Q‐type calcium channel activation mediates beta/gamma frequency oscillatory activity in all PPN neurons (Kezunovic et al. 2011). We studied intrinsic membrane oscillations in 27 PPN neurons using 1 sec long depolarizing current ramps, in the presence of SBs and TTX. Depolarizing 1 sec current ramps were used to determine the voltage dependence of their oscillatory behavior as previously described (Kezunovic et al. 2011, 2013). Since our previous findings showed that PPN neurons cannot be effectively depolarized beyond −25 mV using square steps due to the activation of K^+^ channels during rapid depolarization (Kezunovic et al. 2011, 2013), we studied the effects of NCS‐1 and Li^+^ using a 1 sec depolarizing ramp, gradually changing the membrane potential from resting values up to 0 mV in current clamp mode, to induce membrane oscillations in all three groups of cells present in the PPN. The protocol applied a 1 sec duration current ramp that reached a maximum of 700 pA, executed shortly after breaking into the cell and every 5 min thereafter, for up to 30 min.

**Figure 1:**
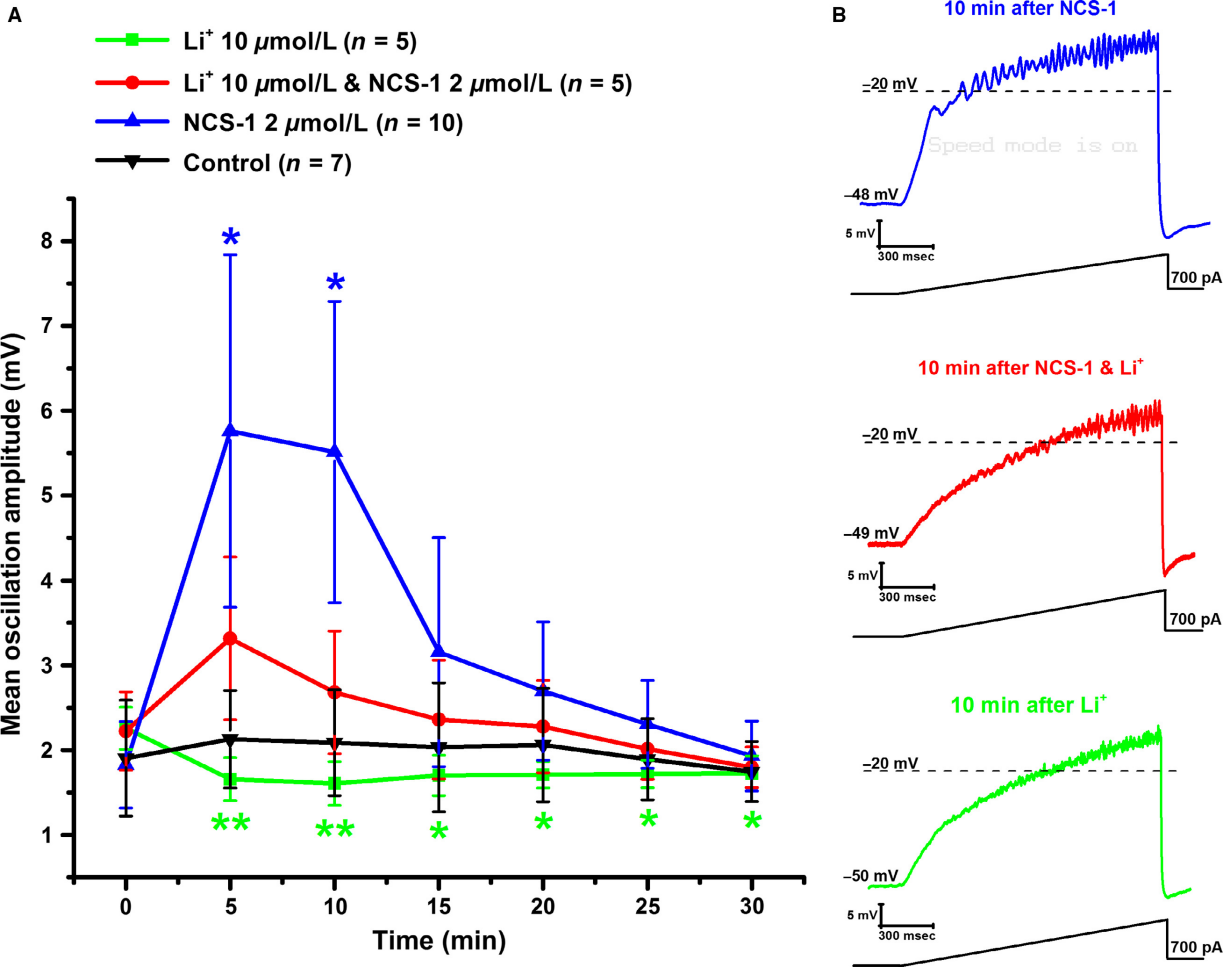
Effects of NCS‐1 and Li^+^ on ramp‐induced oscillations in PPN neurons. (A) Graph of mean oscillation amplitude for groups of cells exposed to no agents or Control (Black inverted triangles, *n* = 7), those exposed to NCS‐1 at 2 μmol/L in the pipette (Blue triangles, *n* = 10), those tested with Li^+^ at 10 μmol/L in the bath (Green squares, *n* = 5), and those exposed to both NCS‐1 at 2 μmol/L in the pipette and Li^+^ at 10 μmol/L in the bath (Red circles, *n* = 5). Mean and SE of the oscillation amplitudes are plotted as 1 sec ramps were applied as soon as the patch was established at 0 min and every 5 min thereafter until 30 min. Statistical significance at *P* < 0.01 is shown as two asterisks, and at *P* < 0.05 as one asterisk. Briefly, no rundown in oscillation amplitude is observed during the period tested in Control cells, NCS‐1 significantly increased oscillation amplitude at 5 min and 10 min, while Li^+^ decreased oscillation amplitude especially at 5 min and 10 min, and less so after that time, and the combined application produced no significant changes in oscillation amplitude, suggesting that NCS‐1 and Li^+^ had competing effects. (B) Representative ramp‐induced oscillations. Top record (Blue) shows the effects of a ramp applied 10 min after exposure to NCS‐1 in the pipette on a PPN neuron. Middle record (Red) shows the effects of a ramp applied 10 min after exposure to NCS‐1 and Li^+^ on a PPN cell. Bottom record (Green) shows the effects of a ramp applied 10 min after exposure to Li^+^ on a PPN neuron.

A group of control neurons (*n* = 7) were patched using normal intracellular recording solution and tested using 1 sec ramps applied upon patching and every 5 min for 30 min. The average amplitude (2.0 ± 0.5 mV) of the oscillations was similar to those observed in previous studies in the absence of stimulation with carbachol or modafinil (Kezunovic et al. 2011, 2013; D'Onofrio et al. 2015). As previously observed, beta/gamma oscillations were present without rundown of high threshold, voltage‐dependent calcium channel mediated responses. Using repeated measures ANOVA, we determined that the amplitude of the ramp‐induced oscillations at min 0 (zero) were not statistically different from those of the subsequent ramps at 5 min through 30 min (Repeated Measures ANOVA, *df* = 6, *F* = 0.1766, *P* = NS) in control cells. Mean peak oscillation amplitude was measured by taking the mean of the three consecutive peak amplitude oscillations in each ramp after filtering. Figure 1A shows that in control cells (black inverted triangles), the mean oscillation amplitude (2.0 ± 0.5 mV at 0 min) remained close to that amplitude for 30 min. We then tested the amplitude of ramp‐induced oscillations at min 0 in the control cells against each of the subsequent groups of cells in which NCS‐1 and/or Li^+^ was present in the pipette at min 0. The amplitude of oscillations were not statistically different between min 0 in control cells and each min 0 recording with NCS‐1 and/or Li^+^ present in the pipette (*df* = 3, *F* = 0.064, *P*=NS for ANOVA). Therefore, we concluded that the min 0 recordings with intracellular NCS‐1 and/or bath applied Li^+^ were indeed similar to control recordings.

### Effects of NCS‐1 on the oscillatory activity of PPN neurons

We previously reported that NCS‐1 at 1 μmol/L concentration decreased the amplitude of these oscillations, but at ~20 min latency (D'Onofrio et al. 2015). A group of neurons were patched to study the effects of NCS‐1 at 2 μmol/L concentration (*n* = 10). This concentration induced increases in oscillation amplitude at shorter latencies, 5 min and 10 min, than lower concentrations. Figure 1A shows the mean oscillation amplitude of these cells after 5 min and every 5 min thereafter for 30 min (blue triangles). Figure 1A shows that mean oscillation amplitude using 2 μmol/L NCS‐1 at the beginning of recording (0 min) was 1.8 ± 0.5 mV, which increased significantly to 5.8 ± 2 mV after 5 min, and to 5.5 ± 1.8 mV at 10 min (Repeated Measures ANOVA, *df* = 6, *F* = 3.08, *P* < 0.02 for ANOVA; Bonferroni post hoc test, at 5 min, *t* = 3.0, *P* < 0.03; and 10 min, *t* = 2.8, *P* < 0.05). Figure 1B shows an example of ramp‐induced membrane potential oscillations in a PPN neuron using a 1 sec ramp protocol after 10 min of NCS‐1 at 2 μmol/L, which was shown to significantly increase the amplitude of oscillations (top record, blue). The effects of NCS‐1 at 2 μmol/L suggest that the modulation of Ca^2+^ channel activation of PPN neurons promoted gamma band oscillation amplitudes at short latency.

### Effects of Li^+^ on the oscillatory activity of PPN neurons

Our previous findings had tested high‐Li^+^ concentrations at 1 mmol/L, and 10 mmol/L, which rapidly and permanently reduced oscillation amplitudes (D'Onofrio et al. 2015). Here, we tested the effects of Li^+^ at a lower, more, physiological concentration of Li^+^ (10 μmol/L) than we had previously tested. We used a concentration of Li^+^ at 10 μmol/L (*n* = 5) that reached maximum effect when it was bath applied at 5 min after rupturing the membrane and gaining stable access to the intracellular compartment of PPN cells. Statistically significant reductions in oscillation amplitude were observed throughout the recording period. Figure 1A shows that Li^+^ at 10 μmol/L induced a highly significant reduction in mean oscillation amplitude at 5 min (1.7 ± 0.3 mV) and 10 min (1.7 ± 0.2 mV) compared to 0 min (2.3 ± 0.3 mV), with a decrease in the effect after 10 min but still significant (Repeated Measures ANOVA, *df* = 6, *F* = 3.68, *P* < 0.01; Bonferroni post hoc test, for 5 min, *t* = 3.7, *P* < 0.01; for10 min, *t* = 4.0, *P* < 0.01; for 15 min, *t* = 3.4, *P* < 0.02; for 20 min, *t* = 3.4, *P* < 0.02; for 25 min, *t* = 3.3, *P* < 0.02; for 30 min, *t* = 3.3, *P* < 0.02). Figure 1B shows an example of representative ramp‐induced membrane potential oscillations observed in a PPN neuron using a 1 sec ramp protocol after 10 min of bath applied Li^+^ at 10 μmol/L concentration, which were significantly reduced (bottom record, green). Bonferroni post hoc tests revealed significant differences when comparing 0 min to every following time point. These results suggest that Li^+^ has the ability to diminish Ca^2+^ channel‐mediated gamma oscillation amplitude.

### Effects of Li^+^ on NCS‐1 in PPN neurons

In order to test the effects of Li^+^ on NCS‐1, immediately after rupturing the membrane and gaining stable access to the intracellular compartment of the cell, Li^+^ (10 μmol/L) was bath applied, while NCS‐1 (2 μmol/L) was intracellularly applied to the neuron. Figure 1A is a graph showing the mean peak amplitude of oscillations in neurons recorded with NCS‐1 alone (blue triangles), Li^+^ alone (green squares), a control group (black triangles), and in neurons in the presence of both NCS‐1 and Li^+^ (red circles). Figure 1B shows an example of ramp‐induced membrane oscillations after 10 min of intracellular NCS‐1 along with extracellular Li^+^ (middle record, red). The addition of Li^+^ (10 μmol/L) to the extracellular solution was observed to antagonize the effects of NCS‐1 on oscillatory activity in PPN neurons. As described above, NCS‐1 alone significantly increased oscillation amplitude at both the 5 and 10 min time points (blue triangles). This result suggests that NCS‐1 at 2 μmol/L concentration is able to increase gamma oscillation amplitude within 5–10 min. However, the addition of bath applied Li^+^ was shown to blunt the effects of NCS‐1 on oscillatory activity. NCS‐1 was unable to significantly increase the amplitude of oscillations in the presence of Li^+^ (red triangles). Mean oscillation amplitude with 2 μmol/L NCS‐1 and Li^+^ at the beginning of recording (0 min) was 2.2 ± 0.5 mV, which increased slightly to 3.3 ± 1 mV after 5 min, followed by a gradual decrease to 2.3 ± 0.5 mV at the 20 min time point and a further decrease, marginally below initial levels, at the 30 min to 1.8 ± 0.2 mV (*df* = 6, *F* = 2.07, *P*=NS for Repeated Measures ANOVA). These results suggest that this concentration of Li^+^ has the ability to reduce the enhancing effect of NCS‐1 on gamma oscillation amplitude, thereby decreasing Ca^2+^ channel‐mediated oscillations below initial levels.

## Discussion

Briefly, these results show that, (1) NCS‐1 at 2 μmol/L was effective at short latency in reducing the amplitude of ramp‐induced oscillations in PPN neurons, (2) Li^+^ at more physiological concentrations (10 μmol/L) caused a short latency decrease in the amplitude of Ca^2+^ channel‐mediated, high‐frequency oscillations in PPN neurons, and (3) Li^+^ significantly reduced the enhancing effect of NCS‐1 on these oscillations. We previously showed that low concentrations of NCS‐1 increased the amplitude of gamma oscillations, but high concentrations ultimately reduced their amplitude (D'Onofrio et al. 2015). From a clinical standpoint, NCS‐1 over expression in at least some bipolar disorder patients (Koh et al. 2003), would be expected to increase high‐frequency activity initially as concentrations rose, essentially inducing increased arousal and hypervigilance, but as NCS‐1 concentrations continue to rise, these would ultimately block the generation of high‐frequency oscillations. This suggests that an optimal level of NCS‐1 for normal function is essential. The same may be true for Li^+^.

Our current results show that, even at the low concentration tested, Li^+^ reduced high‐frequency oscillations by modulating NCS‐1 and Ca^2+^ concentrations. Li^+^ has been suggested to have both neuroprotective effects and also neurotoxic consequences. Its ability to modulate Ca^2+^ concentrations may be at the root of its neuroprotective effects (Forlenza et al. 2014). Thus, use of this agent would tend, in a normal individual manifesting high‐frequency oscillation during the alerted states of waking and REM sleep, to reduce arousal and/or REM sleep drive, accounting for the soporific effects of the agent. This theory is supported by evidence that Li^+^ treatment has been seen to increase slow wave sleep and reduce REM sleep (Friston et al. 1989; Zamboni et al. 1999; Qureshi and Lee‐Chiong 2004; Jones et al. 2008; Ota et al. 2013), as well as increase REM sleep latency (Campbell et al. 1989). Moreover, in bipolar disorder patients, a common symptom is insomnia (Berger et al. 2003), which is an overactive waking system intruding into sleep time (Garcia‐Rill 2015). Insomnia seen in bipolar disorder could be a result of slightly increased levels of NCS‐1 that lead to increased high‐frequency activity and ultimately, hyperarousal of the waking system. Li^+^ may be able to alleviate the hyperarousal by down regulating the interaction between NCS‐1 and InsP3, thus decreasing high‐frequency activity. The down regulation by Li^+^ of NCS‐1 interactions with InsP3 may reduce the abnormal high‐frequency activity and restore proper rhythms to these systems.

In the case in which NCS‐1 is over expressed, Li^+^ can be expected to downregulate the inhibitory effects of excess NCS‐1, perhaps allowing oscillations to be manifested instead of being inhibited, and thus restoring normal function. Li^+^ blunted NCS‐1‐mediated enhancement of high‐frequency oscillations (Fig. 1) such as those previously described in PPN neurons (D'Onofrio et al. 2015). This suggests that, in individuals over expressing NCS‐1, Li^+^ would down regulate NCS‐1 and perhaps restore gamma band oscillations. Although others have described how therapeutic levels of Li^+^ can inhibit the biochemical interaction between NCS‐1 and InsP_3_R (Schlecker et al. 2006), our study identifies one physiological mechanism of Li^+^ action as a key regulator of neuronal high‐frequency rhythmicity in bipolar disorder. This mechanism, reduction of gamma oscillations, accounts for some of the symptoms of the disease. Thus, Li^+^ may be effective not only when NCS‐1 is only mildly increased, but also when over expressed. Such mechanisms might be a part of a wider intracellular cascade which affect a number of possible downstream targets, ultimately leading to an overall down regulation of high‐frequency oscillations mediated by Ca^2+^ channels. NCS‐1 is known to interact with many other target proteins in the brain, including PtdIns 4‐kinase (Hendricks et al. 1999; Rajebhosale et al. 2003), dopamine D2 receptors (Kabbani et al. 2002; Saab et al. 2009; Lian et al. 2011), as well as voltage‐gated Ca^2+^ (Weiss and Burgoyne 2001; Tsujimoto et al. 2002; Rousset et al. 2003) and K^+^ channels (Nakamura et al. 2001; Guo et al. 2002). To further substantiate the view that Li^+^ is specifically disrupting NCS‐1 interaction with InsP_3_R, future studies could determine whether known inhibitors of InsP_3_R also produce a similar reduction in the oscillation amplitude as seen in Figure 1. If the InsP_3_R inhibitors do indeed mimic the effect of Li^+^, then this would more directly connect the observations to a possible regulatory effect of InsP_3_R. Further investigations could clarify the intracellular mechanisms of Li^+^ involved in the treatment of mood disturbances seen in bipolar disorder patients.

On the other hand, Li^+^ can be toxic at high levels, and the potentially fatal Li^+^ toxicity effects on cardiac function could be a result of the interaction between Li^+^, NCS‐1 and InsP3. Notably, NCS‐1 is mainly expressed in the brain and heart (Weiss and Burgoyne 2001; Nakamura et al. 2003). NCS‐1 has been identified as a novel regulator of cardiac Ca^2+^ signaling. Furthermore, a study on immature hearts found that NCS‐1 physically and functionally interacts with InsP3. Stimulation of InsP3 resulted in phosphorylation of CaMKII‐*δ*, which was enhanced by NCS‐1 over expression. These results indicate that a functional link exists between NCS‐1, InsP3 function, and CaMKII activation that potentially affect global Ca^2+^ signals (Nakamura et al. 2011). Li^+^ intoxication can result in cardiac conduction disturbances (Delva and Hawken 2001), and heart disease increases the risk of developing Li^+^ intoxication (Numata et al. 1999; Tielens et al. 1999). It is possible that too much Li^+^ disrupts normal Ca^2+^ signaling and activity, thus causing physiologic and electrochemical changes in the heart leading to abnormal rhythms in both the heart and the brain. Therefore, we speculate that Li^+^ at optimal therapeutic levels will restore proper rhythmicity by inhibiting the effects of NCS‐1/InsP3 pathways, however, too much will result dysfunctional activity.

In summary, Li^+^ treatment would compensate the effects of over expression of NCS‐1 (Koh et al. 2003), and of the reduced gamma (Ozerdem et al. 2011) observed in some bipolar disorder patients, perhaps by partially preventing the action of excessive NCS‐1 and restoring intracellular pathways mediating normal gamma band activity.

## Conflict of Interest

None declared.
